# Optimizing Hyperparameter Tuning in Machine Learning to Improve the Predictive Performance of Cross-Species N6-Methyladenosine Sites

**DOI:** 10.1021/acsomega.3c05074

**Published:** 2023-10-13

**Authors:** Nguyen Quoc Khanh Le, Ling Xu

**Affiliations:** † Professional Master Program in Artificial Intelligence in Medicine, College of Medicine, Taipei Medical University, Taipei 110, Taiwan; ‡ Research Center for Artificial Intelligence in Medicine, Taipei Medical University, Taipei 110, Taiwan; § AIBioMed Research Group, Taipei Medical University, Taipei 110, Taiwan; ∥ Translational Imaging Research Center, Taipei Medical University Hospital, Taipei 110, Taiwan; ⊥ NUS-ISS, National University of Singapore, Singapore, 119615, Singapore

## Abstract

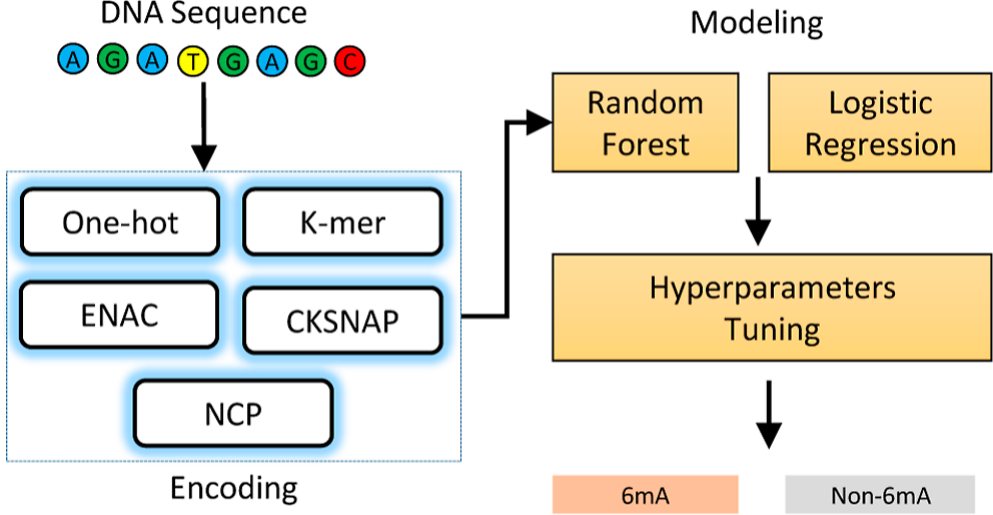

DNA *N*^6^-methyladenosine (6 mA) modification carries significant epigenetic information and plays a pivotal role in biological functions, thereby profoundly impacting human development. Precise and reliable detection of 6 mA sites is integral to understanding the mechanisms underpinning DNA modification. The present methods, primarily experimental, used to identify specific molecular sites are often time-intensive and costly. Consequently, the rise of computer-based methods aimed at identifying 6 mA sites provides a welcome alternative. Our research introduces a novel model to discern DNA 6 mA sites in cross-species genomes. This model, developed through machine learning, utilizes extracted sequence information. Hyperparameter tuning was employed to ascertain the most effective feature combination and model implementation, thereby garnering vital information from sequences. Our model demonstrated superior accuracy compared to the existing models when tested using five-fold cross-validation. Thus, our study substantiates the reliability and efficiency of our model as a valuable tool for supplementing experimental research.

## Introduction

*N*^6^-methyladenosine (6 mA) refers to the methylation of the adenosine base at the nitrogen-6 position, initially identified in the 1970s.^1,2^ As a distinct epigenetic marker,^3,4^ 6 mA plays a vital role in various biological processes including but not limited to gene expression regulation, DNA damage repair, and viral infection. This modification is observed across a broad range of species, encompassing viruses, mammals, eukaryotes, insects, and plants.^5^ Intriguingly, 6 mA modifications have been linked to multiple diseases such as obesity, cancer, and neurodegenerative disorders, including Parkinson’s and Alzheimer’s.^6,7^ Thus, the accurate identification of 6 mA sites could have a profound influence on unraveling associated mechanisms and exploring related biological processes.

Experimental and computational methods are typically employed to analyze the presence of 6 mA sites. For instance, McIntyre et al. utilized Methylated RNA Immunoprecipitation Sequencing (MeRIP-seq) for mapping 6 mA and detecting its variations.^8^ Similarly, a photo-cross-linking-assisted 6 mA sequencing strategy (PA-m6A-seq) was employed to establish a human 6 mA map and reveal novel modification sites.^9^ Despite their direct exploration of 6 mA modification, these experimental methods can be costly and time-intensive, particularly for high-throughput sequencing.^1^ As a result, machine-learning-based computational approaches have been developed to predict 6 mA sites with high accuracy. These models leverage a variety of features such as sequence context, physicochemical properties, and evolutionary conservation to distinguish between 6 mA and non-6 mA sites. For example, Chen et al. incorporated nucleotide chemical property and accumulated nucleotide frequency information as feature descriptors and employed a support vector machine (SVM)-based method for 6 mA prediction.^9^ Similarly, Liu et al. implemented one-hot encoding and devised the im6A-TS-CNN method using a convolutional neural network (CNN).^10^ Another CNN-based methodology was developed by Alam et al., integrating one-hot encoding with nucleotide chemical properties.^11^ Moreover, Abbas et al. applied one-hot encoding for genome data transformation and developed a deep neural network (namely, TS-m6A-DL) to classify 6 mA sites in humans, mice, and rats.^6^ Subsequent studies have led to the development of other genome 6 mA site identification tools such as MethyRNA,^12^ M6AMRFS,^13^ Le and Ho,^14^ i6 mA-Caps,^15^ ENet-6 mA,^16^ m6A-TSHub,^17^ DeepM6ASeq-EL,^18^ and I-DNAN6 mA.^19^

The successful development of machine learning models for 6 mA prediction significantly facilitates our understanding of its functional roles and molecular mechanisms in various biological processes, thereby providing valuable insights into the development of novel therapeutic strategies. Inspired by previous successful research, we aim to further investigate various encoding methods and models for detecting 6 mA sites. This study utilizes computational methods for the identification of 6 mA sites, employing a diverse range of encoding methods for the DNA and RNA sequences of various species. While one-hot encoding is the most commonly employed method in the existing studies, we will venture into less-explored encoding techniques. We have also observed that many studies tend to focus on a single model; hence, we intend to examine multiple models and juxtapose their performances. We will also explore the effect of different encoding schemes on different models to understand their effect on model performance. Consequently, this study is guided by two principal objectives: (1) to encode 6 mA data using an array of DNA and RNA encoding schemes and (2) to construct binary classification models for the identification of 6 mA sites.

## Materials and Methods

### Data Transformation

The data set employed in this study was derived from the TS-m6A-DL study,^6^ encompassing DNA or RNA sequence data from four distinct species. These species include *Arabidopsis thaliana*, a small flowering plant; *Homo sapiens*, known commonly as humans; *Mus musculus* or house mouse; and *Saccharomyces cerevisiae*, a yeast species. To construct a high-quality data set, they applied specific criteria such as

- Sequences with a length of 41 nucleotides (nt) that contain the m6A site in the middle were used as positive sequences.
- Sequences with over 80% similarity were removed to eliminate duplication and homology bias, utilizing the CD-HIT software.
- Negative sequences were experimentally validated as nonmethylated for the mentioned tissues. They also have a length of 41 nt and contain Adenine in the center. These sequences exhibit the m6A consensus motif but were not enriched in m6A analysis. For each species, the data set contains an equal number of positive and negative sequences.

**Figure 1:**
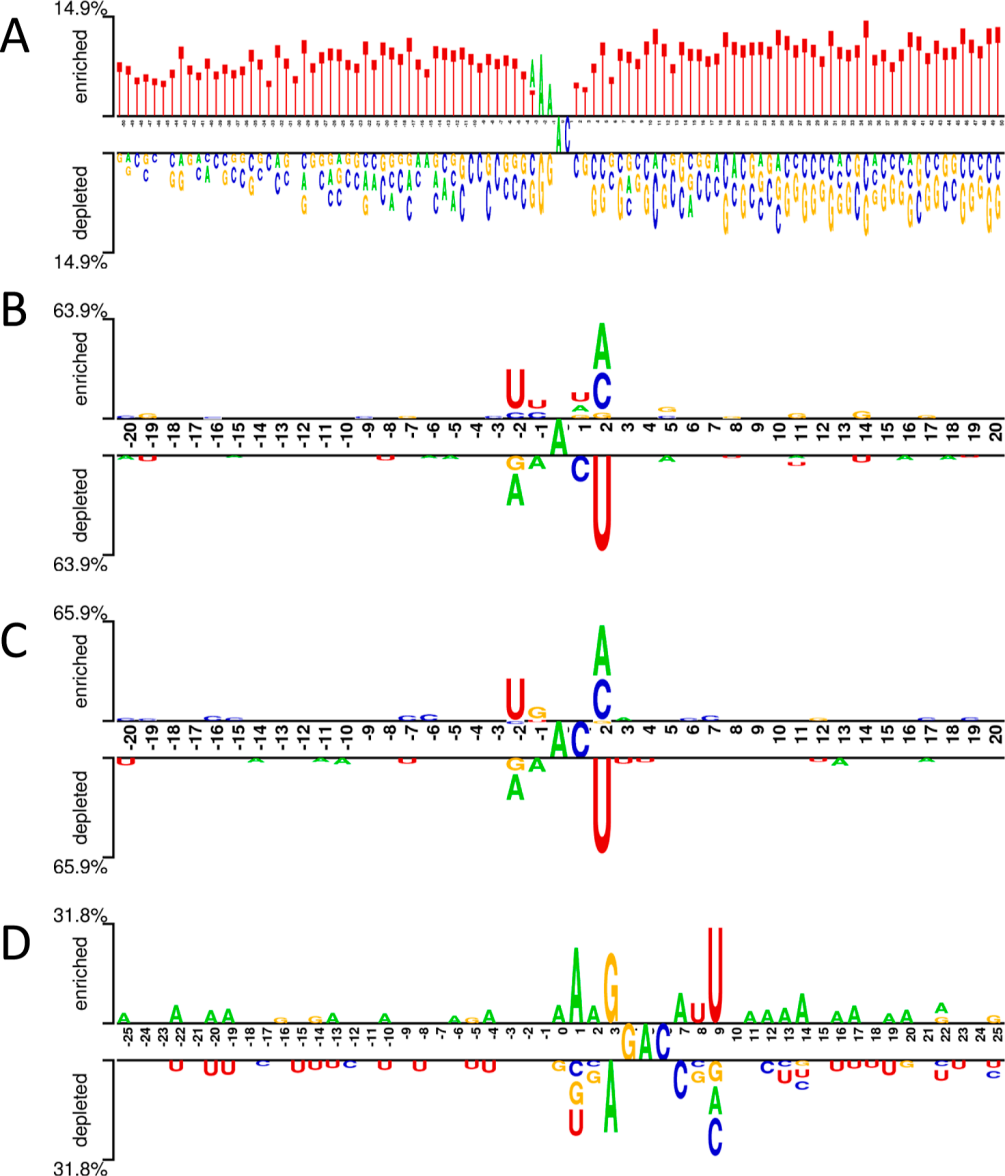
Nucleotide distribution of sequencing data. (A) *Arabidopsis thaliana*, (B) *H. sapiens*, (C) *M. musculus*, and (D) *Saccharomyces cerevisiae*.

To prevent bias toward one class (positive or negative), an equal number of random negative sequences were retrieved to balance the data set, mirroring the number of positive sequences. Figure 1 presents the distribution of nucleotides for each species’ sequencing data with sliding window lengths of 50 for *A. thaliana*, 20 for both *H. sapiens* and *M. musculus*, and 25 for *S. cerevisiae*. To facilitate data encoding, we initially transformed the data files to the FASTA format. A subset of data from the four species is presented in this study, with comprehensive data details summarized in Table 1.

**Table 1:** Data Summary (Number of Sequencing Data Used in This Study)^a^

species	6 mA	non-6 mA	data example
Arabidopsis thaliana	2100	2100	ATAAGAGAAAAGAAAACCTT
H. Sapiens	1130	1130	CUGUGACCUUACAGCUGAGA
M. musculus	725	725	UCCCUGUGAGGAGCAGGGGG
Saccharomyces cerevisiae	1307	1307	GAGUUGAAGUAAAAAUAAAG

a Benchmark data set was collected from the TS-m6A-DL study.^6^

### Encoding Methods

In this study, we utilized iLearn Python toolkit,^20^ which provides methods to encode the genome sequences. We first chose five encoding methods in an attempt. The methods described by Chen et al.^20^ are as follows.

- One-hot encoding (binary): The bases A C G U or T would be represented by 0 and 1. The encoding example is shown below
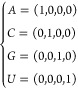
1
- Kmer: This method uses the occurrence frequencies of *K* neighboring nucleic acids. The frequency of Kmer type *t* is calculated as the number of Kmer type *t* appeared in the sequence divided by the total number of occurrences of all Kmer types, where *N*(*t*) is the number of Kmer type *t*.
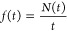
2
- Enhanced nucleic acid composition (ENAC): The frequency of each nucleic acid type in a nucleotide sequence based on the window of fixed length sequence. At the beginning of the sequence, with fixed length would be calculated. By sliding this window from the beginning to the end of the sequence, the frequency of each nucleic acid type in each of the window could be obtained.
- Composition of k spaced nucleic acid pairs (CKSNAP): Frequency of nucleic acid pairs separated by any k nucleic acid. There are 16 types of nucleic acid pair composition for each *k*-space (“A···A”, “A···C”, “A···G”, “A···T”, “C···A”, “C···C”, “C···G”, “C···T”, “G···A”, “G···C”, “G···G”, “G···T”, “T···A”, “T···C”, “T···G”, “T···T”). For example, when *k* = 0, we calculate it as follows
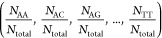
3where *N*_AA_ is the number of times nucleic acid pair AA appears and *N*_total_ is the total number of 0-spaced nucleic acid pairs in the nucleotide sequence.
- Nucleotide chemical property (NCP): Different nucleotides have different chemical structures, so 0 and 1 would be assigned according to each nucleotide’s property and three coordinates are used to represent the chemical properties of the nucleotide. Hence, A = (1, 1, 1), C = (0, 1, 0), G = (1, 0, 0), U = (0, 0, 1).

For a thorough comparison of different encoding schemes, specific values for the Kmer, ENAC, and CKSNAP methods were chosen. These include one-hot encoding, Kmer with a series of *k* neighboring nucleic acids set at 2, 3, 4, and 5; ENAC using a sliding window length of 2, 3, 4, and 5; CKSNAP with k nucleic acid values at 1, 2, 3, 4, and 5; and the application of the NCP method. In total, we implemented 15 unique encoding schemes to encode data from each of the four species.

### Modeling and Analysis

Upon transforming the data into a numerical form using the 15 encoding schemes, we proceeded to apply two models. Initially, a logistic regression model, not typically utilized for such a task due to its simplicity, was constructed. Subsequently, we employed the nonparametric random forest algorithm, which, given its flexibility regarding the mapping function form, anticipated superior predictive performance compared to logistic regression. All models were developed using the Python programming language on a workstation equipped with an Intel core i9-10900X CPU and an NVIDIA GeForce RTX 3080 GPU. Considering the relatively small data sets—with the exception of the *A. thaliana* data set, which contained around 2000 sequences—we validated the models via a five-fold cross-validation process. We used five evaluation metrics—accuracy (Acc), sensitivity (Sens), specificity (Spec), Matthews correlation coefficient (MCC), and area under the curve (AUC)—to assess model performance.^21^
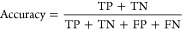
4
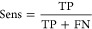
5
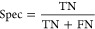
6
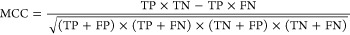
7

## Results and Discussion

### Initial Model Results

**Figure 2:**
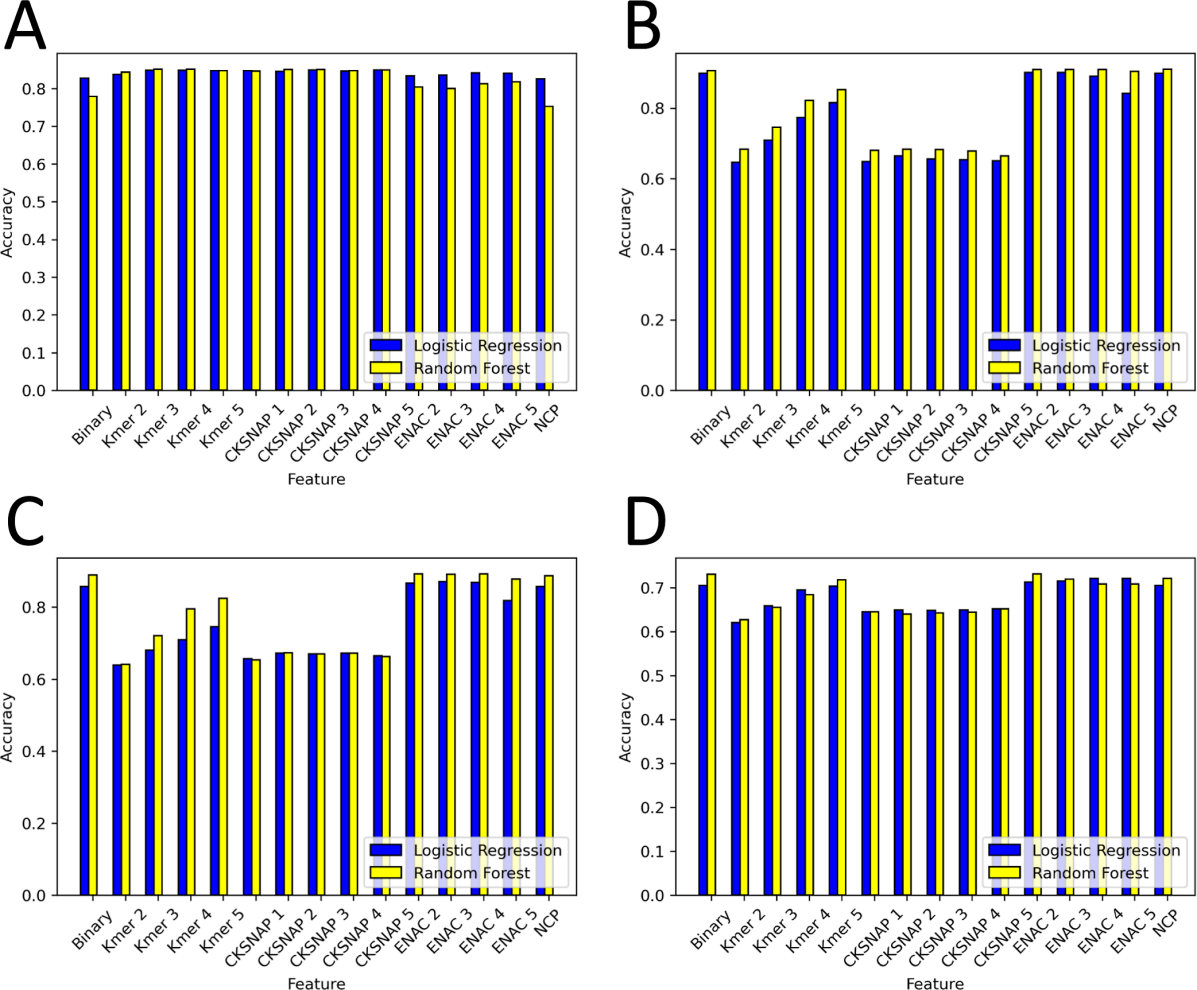
Performance results of machine learning models on different species. (A) *Arabidopsis thaliana*, (B) *H. sapiens*, (C) *M. musculus*, and (D) *Saccharomyces cerevisiae*.

The findings from the models, operating under default parameters and validated via five-fold cross-validation, are detailed in this section, with accuracy visualized in Figure 2. Models employing the CKSNAP-3 and CKSNAP-5 encoding methods consistently produced the highest accuracy (0.849) and MCC values (0.698). The Kmer-5 method yielded the peak AUC and specificity scores (0.911 and 0.87, respectively), while the ENAC-4 method registered the highest sensitivity score (0.845). In general, the performance associated with the NCP method was comparatively weaker.

Examining the random forest models reveals that those built with Kmer-3 and −4 notched the highest accuracy (0.851). The models using CKSNAP-1, -4, and -5 encoding achieved the peak AUC (0.918), while Kmer-5 encoding resulted in the greatest sensitivity (0.867). Additionally, CKSNAP-1 registered the most noteworthy specificity (0.85) and Kmer-4 recorded the highest MCC value (0.706).

The *H. sapiens* model achieves its highest accuracy from the ENAC-2 and ENAC-3 models (0.901). In contrast to the results observed in *A. thaliana*, the Kmer and CKSNAP methods perform less effectively in predicting the 6 mA data of *H. sapiens*. Upon examination of the random forest model, we note that all accuracies surpass those from logistic regression models. Remarkably, the model based on the NCP method records the highest accuracy, specificity, and MCC values (0.91, 1, and 0.834, respectively). Similarly, lower scores emerge from the Kmer and CKSNAP methods. Interestingly, the perfect specificity scores of 1 indicate that the ENAC-2, -3, and NCP models are capable of correctly identifying all non-6 mA sites.

Similar to the models designed for *H. sapiens*, the Kmer and CKSNAP methods yield lower scores in the *M. musculus* model. The binary and NCP methods offer the highest sensitivity values (0.793), while the model constructed using the ENAC-3 scheme delivers the best accuracy (0.871), AUC (0.899), specificity (0.95), and MCC scores (0.752). As for the random forest model, it exhibits superior performance compared with the logistic regression model. The highest accuracy is observed in models utilizing ENAC-2 and ENAC-4 (0.892), with ENAC-3 achieving the highest specificity (1) and MCC scores (0.804). Additionally, the model incorporating ENAC-4 delivers the best AUC (0.91) and sensitivity (0.785) values.

In the *S. cerevisiae* model, the ENAC methods display a superior overall performance, with the ENAC-4 model securing the highest accuracy (0.721) and MCC (0.442) values. As seen in *H. sapiens* and *M. musculus* models, the Kmer and CKSNAP methods yield suboptimal results in comparison to other encoding methods. Notably, the performance for *S. cerevisiae* falls short compared with the other species. Within the random forest model, the ENAC-2 method delivers the best accuracy (0.731), AUC (0.783), and specificity (0.748) scores. The binary method excels in terms of the MCC value (0.465), while the NCP method achieves the top sensitivity score (0.721). Nevertheless, these scores are lower than those observed for other species, indicating a need for model hyperparameter tuning.^22^

In summary, random forest models tend to exhibit superior performance. Diverse descriptors used to represent sequence data yielded varying results across models. For instance, models using the Kmer and CKSNAP methods for *A. thaliana* display elevated scores, whereas the same methods yield diminished scores for other species. Certain encoding methods outperform the binary method, as they potentially encapsulate more DNA or RNA sequence properties. Future work could explore deeper representation learning methods to generate additional features, thereby enhancing predictive performance.^23^

### Tuning Model Hyperparameters

We utilized a randomized search to fine-tune model hyperparameters and obtain optimized models. For logistic regression, we applied a regularization parameter (*C*) of 11.29 and a maximum number of iterations (max_iter) set at 2500 and utilized the “*saga*” solver algorithm. Concurrently, the random forest classifier was configured with the maximum features (max_features) parameter set to “*sqrt*” and a total of 1000 estimators (n_estimators). These meticulously chosen parameter settings were instrumental in achieving optimal model performance for the task of the m6A site prediction, ensuring robustness and reliability in our computational analysis. The results from the tuned models are presented subsequently.

For *A. thaliana*, the Kmer-4 models show the highest accuracy, sensitivity, and MCC (0.867, 0.86, and 0.343, respectively), while Kmer-5 excels in AUC (0.926) and specificity (0.876). In contrast, the NCP method delivers inferior scores. Considering the random forest model, Kmer-4 models boast the highest accuracy, AUC, and specificity (0.861, 0.921, and 0.855, respectively), while Kmer-5 presents the most impressive AUC (0.921) and sensitivity (0.876) values. Again, the NCP method lags behind.

Turning to *H. sapiens*, we found that the binary and ENAC-2 models yield the highest accuracy (0.899), AUC (0.911), and sensitivity (0.821) values. Furthermore, ENAC-2 also displays the greatest specificity (0.997) and MCC (0.809) values. Models utilizing the Kmer and CKSNAP methods continue to underperform. As expected, the random forest model outperforms its logistical counterpart overall. Particularly, ENAC-2 and ENAC-3 models reach the highest accuracy (0.91), specificity (1), and MCC (0.834) values. Notably, the NCP method also exhibits high accuracy (0.91), AUC (0.922), and specificity (1) values. Models built using Binary, ENAC, and NCP achieve a specificity of 1, indicating flawless identification of all non-6 mA sequences. Again, the Kmer and CKSNAP methods show less promising results.

In the *M. musculus* model, the ENAC-4 method delivers the highest accuracy (0.876) and sensitivity (0.794) values, while the ENAC-3 model prevails in terms of AUC (899) and MCC (0.763) scores. Once more, the Kmer and CKSNAP methods are overshadowed by the others. The random forest model employing the ENAC-3 method consistently tops all metrics, with the Kmer and CKSNAP methods falling short.

Finally, with regard to *S. cerevisiae*, we observed relatively suboptimal model performance in this species. The models built using the ENAC methods show superior overall performance, while models employing the Kmer and CKSNAP methods lag behind. Concerning the random forest model, the binary method achieves the highest accuracy (0.737), AUC (0.789), and sensitivity (0.754). Meanwhile, ENAC-2 delivers the highest specificity value (0.734). The binary model also exhibits the highest MCC value (0.475), albeit it is still too low to be considered good.

### Model Comparison

The following graph provides a comparison between the original and optimized models constructed for the four species, each using different encoding methods. In essence, the performance of the models varies depending on the descriptors used to represent the sequence data. In the case of *A. thaliana*, models utilizing Kmer and CKSNAP methods yield superior performances, but these two encoding methods do not fare as well for other species. Clearly, certain encoding methods outperform the binary method, likely due to their ability to consider a more extensive array of DNA or RNA sequence properties.

It is noteworthy that the random forest models, constructed with certain encoding methods, yielded lower scores compared to the logistic regression models for *A. thaliana* and *S. cerevisiae*. This discrepancy could stem from the issue of overfitting. Upon examination of the training scores, we discovered that these training accuracies achieved a perfect score of 1, suggesting an overfitting scenario. To address overfitting, potential solutions include expanding the training data set or reducing the number of features incorporated in the models.

Next, we set our results in the context of previous studies. While there has been substantial prior work on 6 mA identification, we specifically compared our performance against studies that utilized the same data set. Among these, Chen et al.^12^ proposed MethyRNA, which employs the SVM algorithm alongside chemical property and nucleotide frequency as feature descriptors for *H. sapiens* and *S. cerevisiae*. Qiang et al.^13^ proposed M6AMRFS, leveraging an eXtreme Gradient Boosting (XGBoost) model for the four species, featuring dinucleotide binary encoding and local position-specific dinucleotide as feature descriptors. Table 2 presents the model results with our best-performing models for each species also enumerated for comparison.

**Table 2:** Comparison with Previous Studies

species	method	accuracy	sens	spec	MCC
*A. thaliana*	M6AMRFS^13^	0.811	0.807	0.814	0.621
	LR + Kmer #4	0.867	0.860	0.873	0.734
*H. sapiens*	MethyRNA^12^	0.904	0.817	0.991	
	M6AMRFS^13^	0.910	0.820	1	0.834
	RF + ENAC #2	0.910	0.820	1	0.834
M. musculus	MethyRNA^12^	0.884	0.778	1	
	M6AMRFS^13^	0.793	0.828	0.758	0.588
	RF + ENAC #3	0.892	0.785	1	0.804
S. cerevisiae	M6AMRFS^13^	0.743	0.752	0.733	0.485
	RF + binary	0.737	0.754	0.721	0.475

For *A. thaliana*, we opted for the logistic regression model with the Kmer #4 encoding method. This model’s accuracy, sensitivity, and specificity exceeded those of M6AMRFS^13^ by more than 0.05, and its MCC surpassed M6AMRFS by approximately 0.1. For *H. sapiens*, we selected the random forest model with ENAC 2, which showcased comparable performance to M6AMRFS,^13^ while outperforming MethyRNA^12^ by 0.06, 0.03, and 0.09 in terms of accuracy, sensitivity, and specificity, respectively. For *M. musculus*, we chose the random forest model with ENAC #3. Although the sensitivity of this model fell 0.04 short of M6AMRFS,^13^ it outperformed the other two methods in all other metrics. Finally, for *S. cerevisiae*, we employed a random forest model with a binary encoding method. However, this model’s performance was marginally weaker than that of M6AMRFS.^13^ Although the performance results achieved promising results, further works may consider state-of-the-art methods in this field to improve the predictive performance.^24,25^ Sens: Sensitivity, Spec: Specificity; MCC: Matthews correlation coefficient; LR: Logistic Regression; RF: Random Forest.

### Cross-Species Testing

**Figure 3:**
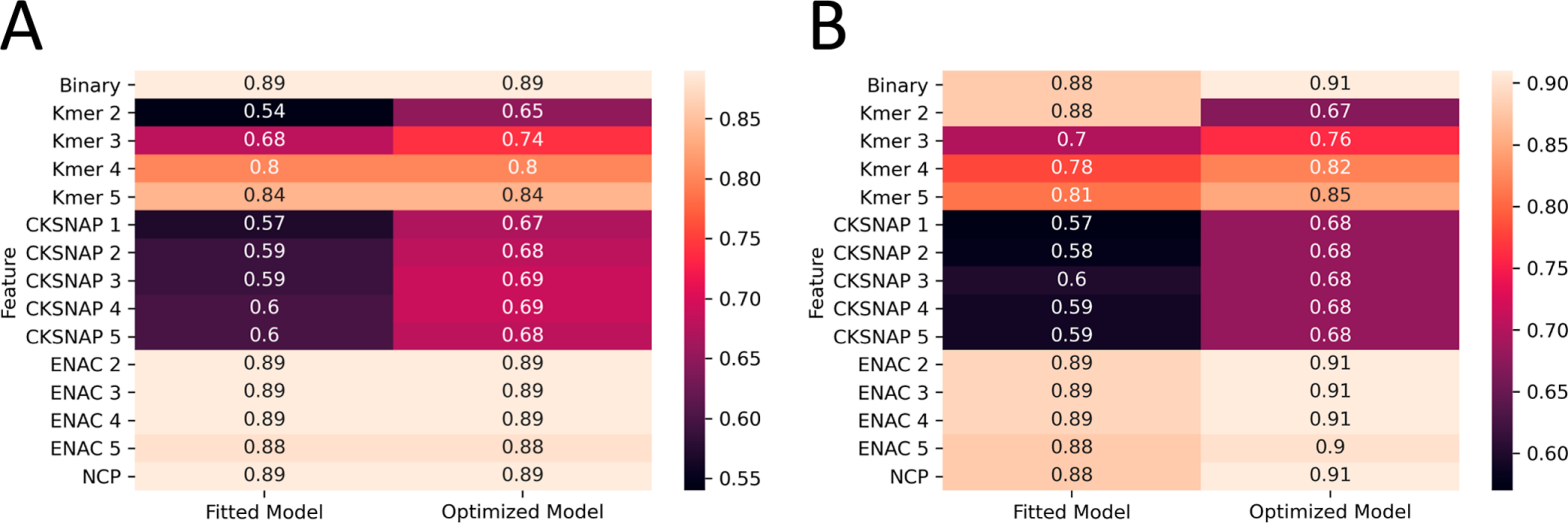
Cross-species testing. (A) Using *H. sapiens* for training and *M. musculus* for testing. (B) Using *M. musculus* for training and *H. sapiens* for testing.

To explore the possibility of utilizing models built for one species to predict others, we could examine the optimized models’ performance on the corresponding encoded data of different species.^26^ This exercise could be beneficial in assessing the models’ generalizability. For instance, we could train the random forest model using *H. sapiens* data and then test it on *M. musculus* data. The process is then repeated in reverse, training the model on *M. musculus* data and testing it on *H. sapiens* data. The testing accuracies derived from this cross-species approach are then juxtaposed with those of the optimized model, as depicted in Figure 3.

When we juxtapose the testing accuracies of *M. musculus* models with the optimized *M. musculus* models, we observe that all testing accuracies are either lower than or equivalent to the optimized accuracies. For models employing binary, ENAC, and NCP encoding methods, there is an almost identical correlation between the testing results and the optimized model. The disparity in accuracy for the other encoding methods does not exceed 0.11.

Upon comparison of the testing accuracy of *H. sapiens* models with their optimized counterparts, we observe that all testing accuracies fall short of the optimized values. The overall discrepancy in accuracy fluctuates between 0.02 and 0.12. Notably, the newly fitted models produced slightly less optimal results. However, when taking into account the minimal variation between cross-species testing accuracy and the optimized model accuracy, along with comparable scores, we can affirm that these models demonstrate satisfactory performance in terms of generalization.

## Conclusions

In this study, we have implemented different machine learning models to predict the presence of 6 mA sites, uncovering that certain encoding methods yield superior results due to their ability to more comprehensively encapsulate the properties of DNA or RNA sequences. Hyperparameter tuning greatly enhanced both models’ performances, with some optimized models rivaling or exceeding the results from prior studies. Interestingly, despite its infrequent use in previous investigations, logistic regression demonstrated commendable performance, yielding straightforward and linear models and presenting itself as a feasible alternative to more complex models. Cross-species testing further revealed that the models optimized for one species maintained their performance when employed for 6 mA site predictions in other species, hinting at their potential broad applicability.

## Data Availability

Our source codes and data sets are freely available at https://github.com/khanhlee/cs6mA-pred.
